# CTTTA Deletion/Insertion polymorphism in 3'-UTR of *LEPR* gene in type 2 diabetes subjects belonging to Kashmiri population

**DOI:** 10.1186/s40200-014-0124-z

**Published:** 2014-12-20

**Authors:** Iqra Hameed, Shariq R Masoodi, Dil Afroze, Riyaz A Bhat, Niyaz A Naykoo, Shahnaz A Mir, Idrees Mubarik, Bashir A Ganai

**Affiliations:** Department of Biochemistry, University of Kashmir, Srinagar, India; Department of Endocrinology, Sher-I-Kashmir Institute of medical Sciences, Srinagar, Kashmir India; Department of Immunology and Molecular Medicine, Sher-I-Kashmir Institute of medical Sciences, Srinagar, Kashmir India; Centre for Research and Development (CORD), University of Kashmir, Srinagar, India

**Keywords:** Polymorphism, Type 2 diabetes mellitus, Kashmir valley, Ethnic population, LEPR

## Abstract

**Background:**

Type 2 diabetes mellitus is a multi-factorial disease in which both genetic and non-genetic factors interact in order to precipitate the diabetic phenotype. Among various predisposing genetic loci, a pentanucleotide (CTTTA) Del/Ins variant in the 3'-UTR of the *LEPR* gene is associated with type 2 diabetes and its related traits. This study was done to explicate for the first time the association of this Del/Ins polymorphism of *LEPR* gene in type 2 diabetes patients belonging to the ethnic population of Kashmir valley.

**Methods:**

670 unrelated subjects comprising of 320 type 2 diabetes patients and 350 healthy controls were included in the study. Genotyping of the untranslated region of *LEPR* gene encompassing this Del/Ins variant was done by PCR-RFLP technique and results were validated by direct sequencing.

**Results:**

Genotype frequencies for both type 2 diabetes cases and healthy controls were consistent with Hardy-Weinberg equilibrium (χ^2^ = 3.09 and 2.37, *P* = NS). The Del/Del genotype was predominantly found in cases than controls (*P =* 0.003, OR: 0.62, CI: 0.45-0.85). Carriers of Ins/Ins genotype were relatively protected against the risk factors (*P =* 0.0004, OR: 0.31, 95% CI: 0.15-0.61). A positive association was observed between the Del allele and the risk factors of type 2 diabetes.

**Conclusion:**

The results elucidate that the CTTTA Del allele is a genotypic risk factor of type 2 diabetes in the Kashmiri population.

## Background

Type 2 Diabetes Mellitus (T2DM) is a multifarious disorder caused by interactions between susceptible genetic loci and environmental slurs. T2DM is an incipient non-communicable global epidemic [1]. The genetics of T2DM is incoherent with respect to the patterns of simple Mendelian inheritance. Contemporary studies inspecting the genetic elements involved in the development of T2DM have not identified any disease-causing mutations; however several predisposing genetic variants (polymorphisms) have been recognized [2–4]. The heterogeneity of this disorder renders the understanding of risk factors and sequence of events obscure. Currently the involvement of diverse proteins produced and secreted by adipocytes, collectively termed as adipokines is recognized to modulate insulin resistance [5]. Amongst these adipokines; leptin, resistin, TNF-α and free fatty acids are implicated in convening insulin resistance whereas adiponectin improves sensitivity. Inconsistency between insulin resistant and insulin sensitizing adipokines is implicated in type 2 diabetes and related disorders. Leptin exerts its central and peripheral effects by binding to its receptors that belong to the class I cytokine receptor superfamily [6]. This binding results in subsequent activation of downstream signalling pathways regulating satiety and stimulating energy expenditure [7]. Sequential phenotypic manifestations of diabetes like hyperinsulinemia, obesity and β-cell failure are observed in animal models (*ob/ob* and *db/db* mice) [8,9]. Disparity between leptin-insulin alliance in pancreatic β-cells as a result of leptin resistance in overweight patients has been suggested to promote hyperinsulinemia and subsequent development into T2DM [10,11]. In pancreatic β-cells, inhibition of insulin secretion by leptin has been shown to be mediated by the long form of *LEPR* gene [12,13]. The human leptin receptor gene (LEPR) is localised in proximity of the microsatellite marker D1S198 which is associated with acute insulin response [14–16]. The 3'-UTR (untranslated region) of LEPR gene encompasses a pentanucleotide (CTTTA) Deletion/Insertion polymorphism. Studies investigating this Del/Ins polymorphism have shown an association with susceptibility to type 2 diabetes and related risk factors [15,17–22]. We screened the CTTTA-pentanucleotide Del/Ins in 3'-UTR of *LEPR* gene in ethnic Kashmiri population of north India. Our study is the first ever molecular characterization of type 2 diabetes mellitus patients of Kashmiri valley aimed to study a possible association between this genetic polymorphism and type 2 diabetes mellitus.

## Methods

### Study population and Clinical parameters

The study included 670 ethnic Kashmiri subjects, comprising of 320 unrelated type 2 diabetes mellitus patients and 350 healthy controls assenting to participate voluntarily. Type 2 diabetes cases were diagnosed as per ADA/WHO criteria. Controls were matched for age and gender. Exclusion criteria involved patients with drug induced, stress induced and gestational diabetes. The study was approved by institutional ethical committee (IEC-SKIMS) and the research protocol conforms to the provisions of the Declaration of Helsinki (as revised in Tokyo 2004). 4 ml of blood was collected from the cubital vein after 12 hours of fast from each subject. Serum was separated from 2 ml of blood and immediately sent for biochemical analyses. Remaining 2 ml of blood was stored in EDTA vacutainer at −70°C until processed for isolation of DNA. Laboratory tests including fasting plasma glucose (FPG), total cholesterol (TC), triglycerides (TG), high density lipoprotein (HDL), low density lipoprotein (LDL) and serum creatinine levels were measured on an autoanalyzer.

Hypertension was defined as a systolic pressure ≥140 mmHg or diastolic pressure ≥90 mmHg. Blood pressure (BP) was measured using standard sphygmomanometer in the sitting position after a 10 minute rest. Anthropometric data comprised of Body Mass Index (BMI) and Waist to Hip ratio (WHR). Height and weight were measured using standard anthropometric techniques in light-weight clothing without shoes. Waist circumference was measured midway between the lower rib margin and the iliac crest at the end of gentle expiration using an anthropometric measuring tape. BMI was expressed as kg/m^2^.

### Genotyping

DNA was extracted from peripheral leukocytes by Qiamp Blood Mini extraction kit (Qiagen Valencia, CA). The variant CTTTA pentanucleotide insertion was screened by polymerase chain reaction (PCR) by amplifying a 114/119 bp region of LEPR gene using 5' mismatch primer ATAATGGGTAATATAAAGTGTAATAGAGTA and 3' primer AGAGAACAAACAGACAACATT in a thermal cycler (Eppendorf Hamburg, Germany). PCR cycling conditions comprised of an initial denaturation for 7 min at 95°C followed by 30 repeats (30 sec each) at 95°C, 55°C and 72°C and a final extension step of 7 min at 72°C. Amplified products were verified by electrophoresis using 2% agarose gel. 10 μl aliquots of the amplified products were digested overnight at 37°C with the restriction enzyme *Rsa I* (Fermentas). Digested products were resolved on 4% agarose gel and cleavage products were visualized by ethidium bromide staining. Deletion genotype was not amenable to restriction digestion and resolved as a single 114 bp band whereas the Insertion genotype was digested by *Rsa I* enzyme yielding 90 bp and 29 bp fragments. Heterozygous Insertion/Deletion genotype was characterized by all three bands of 114 bp, 90 bp and 29 bp. Results were further validated by direct sequencing. Purification and Sequencing was done by Macrogen Inc.1001 World Meridian Venture Center. The genotypic results were in conformity with the sequencing analysis.

### Statistical analyses

Data were managed by Microsoft excel and Java Stat software. Power calculation of the study was performed by online statistical calculator (http://osse.bii.a-star.edu.sg/calculation2.php). The power of study measures 81.4% for observed frequency of minor alleles at 5% significance level. Statistical Package for Social Sciences (SPSS, version 20.0) was used for statistical analysis. Data was described as Mean ± SD and percentage. Chi-square (χ^2^) test and odds ratio analysis were used for genotyping where P value of <0.05 was considered to be significant. Hardy and Weinberg equilibrium was determined by χ^2^ analysis. Inter group variants of metric data was measured by Students t-test at 95% confidence interval. The non-metric data was analyzed by Mann Whitney-U test.

## Results

**Figure 1:**
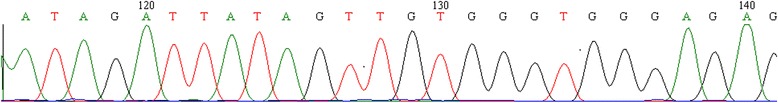
**Partial sequence electropherogram of 3'-UTR Del/Ins polymorphism of** ***LEPR*** **gene (Homozygous wild Del/Del).**

**Figure 2:**
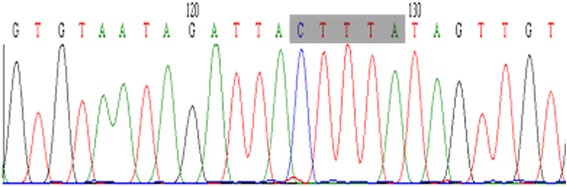
**Partial sequence electropherogram of 3'-UTR Del/Ins polymorphism of** ***LEPR*** **gene (Homozygous variant Ins/Ins).**

**Figure 3:**
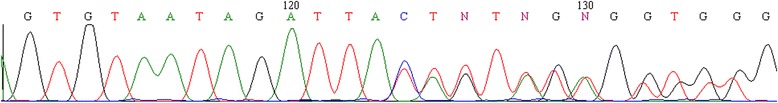
**Partial sequence electropherogram of 3'-UTR Del/Ins polymorphism of** ***LEPR*** **gene (Heterozygous Del/Ins).**

320 unrelated subjects with type 2 diabetes mellitus and 350 healthy controls were screened for the Del/Ins polymorphism in 3'-UTR of the *LEPR* gene. The predominant age group was 50–60 years and the mean age of cases and controls was 50.4 ± 11.1 years and 49.2 ± 12.4 years respectively. 69.5% of cases had a familial history of type 2 diabetes. 55.2% cases were shown to be hypertensive by systemic examination. The status of disease was indicated by HbA1c levels. HbA1c > 8% corresponded to 41% of cases, illustrating uncontrolled diabetes state and improper management of disease in such subjects. Molecular characterization of 3'-UTR of the *LEPR* gene by PCR-RFLP revealed three genotypes (Del/Del, Del/Ins and Ins/Ins) in our population (Figures 1, 2 and 3). Genotypes were in Hardy Weinberg equilibrium for both cases (χ^2^ = 3.03, *P* = Non Sig) and controls (χ^2^ = 2.16, *P* = Non Sig). The homozygous Del/Del genotype was present in 55.6% of cases and 41.7% of controls. 40.4% of cases and 48.6% of controls exhibited the Del/Ins genotype (Table 1). The rare Ins/Ins genotype was present in only 9.7% of controls and 4% of cases. The homozygous Del genotype was significantly higher in the cases than in controls (*P =* 0.003). Frequency of Del allele was 0.75and 0.66, while as the frequency of Ins allele was 0.24 and 0.34 in cases and controls respectively (OR: 0.62, 95% CI: 0.48-0.78). The frequency of Del allele correlated directly with the risk factors and the severity of disease as shown by the intra-group genotype analysis (Table 2). Among the cases, there was a significant association between the presence of Del allele and various risk factors. Del allele was observed to be directly associated with elevated BMI, WHR and total cholesterol. Although fasting and post-prandial glucose levels didn’t vary significantly across Del and Ins genotypes, association of HbA1c levels were statistically significant. This can be due to disparity in the glycemic variability or underlying poor control over longer duration. The glycemic control of the studied population is a major reason for extensive difference between plasma glucose and HbA1c levels. Systemic examination of subjects showed that the carriers of Del allele were primarily hypertensive. Higher incidence of Ins allele in the control population is reminiscent of its protective role against the risk factors of T2DM in our population. The intra-group genotyping analysis in controls showed a linear association of the risk factors with the three genotypes: Del/Del, Del/Ins and Ins/Ins. Del allele was associated with atypical profile of various risk factors that play a role in predisposing the carriers of Del allele to T2DM whereas the Ins allele exhibited classic values for the same in the control population.

**Table 1:** **Genotype analysis and Allele frequency of 3'-UTR Del/Ins polymorphism in** ***LEPR*** **gene**

**LEPR 3'** **-UTR CTTTA polymorphism**	**T2DM cases**		**Controls**		**OR (95% CI)**	**χ** ^**2**^	***P*** **value**
**Genotypes**	**n**	**%**	**n**	**%**			
DD^#^	178/320	55.6	146/350	41.7			
DI	129/320	40.4	170/350	48.6	0.62	8.65	0.003
					(0.45-0.85)		
II	13/320	4	34/350	9.7	0.31	12.23	0.0004
					(0.15-0.61)		
DI+ II	142/320	44.37	204/350	58.28	0.57	12.95	0.0003
					(0.42-0.77)		
**Alleles**							
Del	485/640	75.78	462/700	66	0.62	15.43	0.00011
Ins	155/640	24.21	238/700	34	(0.48-0.78)		

Chi-square analysis is used for genotyping analysis where *P* value of <0.05 at 95% confidence interval was considered to be significant.

DD: Deletion/Deletion, DI: Deletion/Insertion, II: Insertion/Insertion.

^#^Reference genotype.

**Table 2:** **Genotypic correlation of 3'-UTR Del/Ins polymorphism of** ***LEPR*** **gene with various clinical and laboratory features among the study population**

**Parameter**	**Del/Del**	**Del/Ins + Ins/Ins**	***P*** **value**
Age (years)	50.7 ± 10.7	49.0 ± 12.6	0.06
BMI (kg/m^2^)	25.0 ± 5.4	23.4 ± 2.6	0.005
WHR	0.92 ± 0.10	0.88 ± 0.09	0.010
BP (Systolic)	140.5 ± 20.7	130.9 ± 18.4	0.003
BP (Diastolic)	83.0 ± 10.4	81.1 ± 9.6	0.242
BG-F (mg/dl)	212.7 ± 91.4	200.1 ± 43.9	0.183
BG-PP (mg/dl)	303.1 ± 121.7	287.6 ± 62.0	0.230
HbA1c (%)	9.4 ± 2.5	8.6 ± 1.1	0.002
TGs (mg/dl)	225.7 ± 128.6	205.0 ± 43.9	0.082
LDL (mg/dl)	108.8 ± 27.1	103.3 ± 18.9	0.172
HDL (mg/dl)	41.3 ± 9.7	40.3 ± 7.5	0.494
Chol (mg/dl)	182.1 ± 37.7	170.2 ± 32.6	0.041

BMI: Body Mass Index, WHR: Waist to Hip ratio, BP: Blood Pressure, BG-F: Blood Glucose Fasting, BG-PP: Blood Glucose Post Prandial, TGs: Triglycerides, LDL: Low Density Lipoproteins, HDL: High Density Lipoproteins, Chol: Cholesterol.

(Data expressed as Mean ± SD, Non metric data was analyzed by Mann Whitney-U test, *P* value for inter group variants measured by Student’s t-test at 95% confidence interval).

## Discussion

The risk of developing diabetes and related metabolic abnormalities is high in Asian Indians as compared to other ethnic groups and is contributed in part by specific genetic factors determining body fat distribution and glucose metabolism. Distinctive features of “Asian Indian phenotype” include major risk factors responsible for increased predilection towards type 2 diabetes [23–27]. Increased level of adipokines that promote insulin resistance is also an underlying biochemical risk factor [28]. The variance in prevalence rates for different ethnic populations substantiates the evidence for a genetic component involved in the development of T2DM. However, non-genetic factors such as environmental and cultural influences can be moderately ascribed to ethnic variability. Studies have shown that while some genes seem to confer increased susceptibility to diabetes in Indians [29,30]; some protective genes in Europeans do not appear to protect Indians [31]. Kashmir valley is an ethnic region situated in the north India. Very few studies highlighting the prevalence of type 2 diabetes in this region have been undertaken [32,33]. The population also exhibits high frequency of undiagnosed diabetes [32]. Lately an increased trend in the rate of T2DM along with a high prevalence of impaired fasting glycaemia (IFG) especially in young adults was observed [33]. Although the socio-geographic features of this population like being a distinct, cut-off valley from other states and highly endogamous and consanguineous marriages practised here, the place offers an interesting and potential population for conducting genetic studies. However, studies exploring the genetic variations or molecular mechanisms involved in the etiopathogenesis of type 2 diabetes are even meagre for this population [34,35].

The foremost advances linking the genetic basis of T2DM has amassed around 40 predisposing loci [3]. A number of sequence determinants are known to affect mammalian mRNA abundance [36]. Occurrence of multiple copies of the core sequence UUAUUUA(U/A)(U/A), designated as the A + U destabilizing element (AUDE), is known to destabilize a variety of mRNA species [37]. A closely reminiscent sequence of 5'-UACUUUAUA-3' is created in the 3'-UTR of the insertion allele. The presence of extra C in this sequence could probably eliminate the destabilizing effect of the core sequence, nonetheless its role is not identified [17]. The 3'-UTR of *LEPR* gene harbors a pentanucleotide Del/Ins polymorphism that produces an AU-rich sequence capable of affecting the mRNA stability by creating a stem-loop structure [22]. The mechanisms by which stem-loops affect mRNA stability is not completely understood though in many cases these motifs have been shown to bind to regulatory proteins, which in turn could affect the rate of degradation and/or translation of mRNA [36,38].

We undertook this study to elucidate the role of CTTTA pentanucleotide Del/Ins polymorphism in *LEPR* gene in ethnic Kashmiri population. In our population frequency of Del allele was 75.78% and 66% and frequency of Ins allele was 24.21% and 34% in cases and controls respectively. The genotypes for this polymorphism were in Hardy Weinberg equilibrium for both cases and controls. In our study, Del allele showed a linear association with the prevalence of diabetes as well as with the associated risk factors. According to our observations, Ins allele conferred a protective effect against the development of T2DM. The genotype and allele frequency of this polymorphism in our population is comparable to another study in which the carriers of the insertion allele had a 79% reduced risk of T2DM compared with non-carriers in the 4-year follow-up [19]. The Del/Ins variant has been found to be associated with serum insulin levels [17,19], serum high density lipoprotein (HDL)-cholesterol and apolipoprotein A (apo A)-I levels [20], and susceptibility to T2DM [20–22]. In our study the intra group genotyping among both cases and controls explicated a positive correlation between the Del allele and the imperative risk factors that predispose our population to T2DM. Our study attests the salvage role of the Ins allele against the predilection and occurrence of T2DM in our population. The elusive factors of relatively higher proportion of IFG in Kashmiri population [32,33] and the overweight-obesity endemic [39] can in part be explained at the molecular level by the prevalence of a genotypic risk factor (Del allele) that might play a role in inflection and predisposition of its carriers to the T2DM. However other genetic factors may modulate this effect in cumulative or independent manner. The elucidation of these factors in larger samples from our population may substantiate the role of pertinent molecular and genetic factors involved in the pathogenesis and progression to T2DM. This study and its findings may be useful and provide some basic information regarding our understanding of multifaceted pathological interconnections between metabolic legacy, genetic predisposition and contemporary surroundings.
